# Geographic Distribution of Phosphine Resistance and Frequency of Resistance Genes in Two Species of Grain Beetles, *Tribolium castaneum* and *Rhyzopertha dominica*, in North America

**DOI:** 10.3390/insects16080749

**Published:** 2025-07-22

**Authors:** Zhaorigetu Hubhachen, Aaron Cato, Edwin Afful, Manoj Nayak, Thomas W. Phillips

**Affiliations:** 1 Department of Entomology, Kansas State University, Manhattan, KS 66506, USA; zhubhachen@centralstate.edu (Z.H.); ajcato@uada.edu (A.C.); edwin.afful@envu.com (E.A.); 2 Bee Genomics and Molecular Lab, Central States University, Room 251, 1400 Brush Row Road, Wilberforce, OH 45384, USA; 3 Postharvest Commodity Protection, Queensland Department of Primary Industries, Brisbane 4102, Australia; manoj.nayak@dpi.qld.gov.au

**Keywords:** dihydrolipoamide dehydrogenase (DLD), red flour beetle, lesser grain borer, stored grain insects, Bostrichidae, Tenebrionidae

## Abstract

Resistance to the fumigant phosphine (PH_3_) was studied for 28 populations of *Rhyzopertha dominica* from eight states of the USA and four provinces of Canada, as well as for 34 populations of *Tribolium castaneum* from twelve states of the USA and four provinces of Canada, using both a discriminating dose bioassay and molecular marker analysis. We used a molecular marker analysis for a point mutation in the gene that encodes dihydrolipoamide dehydrogenase and facilitates the “strong resistance” phenotype in both species. Our results showed that PH_3_ resistance was correlated with higher frequencies of the strong resistance R allele in both species (R^2^ = 0.59 in *R. dominica* and R^2^ = 0.79 in *T. castaneum*). We also found that recessive R allele frequency did not correlate well with the geographic distribution of the resistant populations of these two species (R^2^ = 0.21 in *R. dominica* and R^2^ = 0.15 in *T. castaneum*). Therefore, populations of both species with higher R allele frequencies had higher resistance levels to PH_3_. Our results showed that the geographic distribution of PH_3_ resistance in both species varied and was not related geographically, but this supports the idea that the adaptive evolution of PH_3_ resistance in these species is caused by selection pressure for their resistance genes.

## Simple Summary

This study reports on the genetically based resistance to the grain fumigant phosphine in two of the most significant grain pests worldwide: the lesser grain borer, *Rhyzopertha dominica*, and the red flour beetle, *Tribolium castaneum*. Our research showed that the distribution of phosphine-resistant insects in the United States and Canada was not related to geographic differences in their collection sites. However, the strong resistance levels to phosphine in these two species were correlated with a higher frequency of the resistance gene in several populations. The higher levels of phosphine resistance in these populations were most likely caused by selection pressure for increases in the phosphine resistance gene.

## 1. Introduction

The lesser grain borer, *Rhyzopertha dominica* (F) (Coleotpera: Bostrichidae), and the red flour beetle, *Tribolium castaneum* (Herbst) (Coleoptera: Tenebrionidae), are two of the most destructive stored-product insect pests, causing a tremendous amount of economic loss to stored grains and grain products worldwide each year. Phosphine gas, hydrogen phosphide (PH_3_), has been used as an effective and widely used fumigant for controlling stored grain insects since 1930. Most likely due to long-term selection pressure, sub-optimal fumigation concentration, and/or fumigation failure, PH_3_-resistant populations of *T. castaneum* and *R. dominica* have been reported in many countries since the 1970s [1,2,3,4,5,6,7,8,9,10,11,12,13,14,15,16]. It is noteworthy that the evolution of PH_3_ resistance in grain beetles from the USA has risen dramatically over recent decades. For example, the known resistance levels to PH_3_ in *T. castaneum* increased from 13% in 1990 to 89% in 2012, whereas those in *R. dominica* increased from 67% in 1990 to 100% in 2012 [3,8]. Two genetically different phenotypes of PH_3_ resistance, “weak” and “strong”, were characterized based on lethal dose ratios relative to the susceptible population of about 30-fold or less for weak resistance and up to several 100- to over 1000-fold for strong resistance in *T. castaneum*, *R. dominica*, and *S. oryzae* [9,17,18,19,20].

Genetic studies of *T. castaneum* and *R. dominica* by Collins et al. [17], Jagadeesan et al. [21], and Schlipalius et al. [18,22] showed that two gene loci, *rph1* and *rph2*, were responsible for the “weak” and “strong” PH_3_ resistance phenotypes, respectively, in these stored-grain pests. The *rph1* locus codes for the weak resistance phenotype, providing moderate resistance to PH_3_, whereas *rph2*, by itself, in beetles lacking *rph1*, confers only very low-level resistance. However, when an individual is homozygous for a resistance allele at *rph1* and is either heterozygous or homozygous for *rph2*, the two loci act synergistically to yield a strong resistance phenotype of many 100s-fold compared to concentrations needed to kill susceptible insects. If that same individual were to have homozygous resistant alleles at both the *rph1* and *rph2* loci, it has a much higher level of resistance, known as the “strong” resistance phenotype.

Although the inheritance of PH_3_ resistance genes and their expression in stored-grain pests have received considerable study with regard to quantitative genetics, the underlying molecular mechanism for phosphine resistance was unclear until findings by Schlipalius et al. [19]. That group determined that the strong PH_3_ resistance phenotype from the *rph-2* locus in *T. castaneum* and *R. dominica* was due to a single amino acid mutation in a core metabolic enzyme, dihydrolipoamide dehydrogenase (DLD). Subsequent work determined that the *rph1* locus codes for a cytochrome b5 fatty acid desaturase (*Cyt-b5-r*) [16]. In our earlier study, we applied the sequence information on PH_3_ resistance genes found in Australian grain beetles [19] and we identified single-nucleotide mutations in the DLD gene in resistant North American populations of *T. castaneum* and *R. dominica*, which then led us to identify a single amino acid mutation in DLD as P45/49S [11]. Furthermore, in the same study, we identified several strongly PH_3_-resistant populations of *T. castaneum* and *R. dominica* using a modified bioassay first developed by the Food and Agriculture Organization (FAO) of the United Nations and the first molecular marker analysis based on P45/49S mutations in both *T. castaneum* and *R. dominica*. We recently identified two different mutations in DLD of the heterozygous individuals of a *T. castaneum* population from Brazil [20]. This result indicated that the molecular basis for PH_3_ resistance in the grain beetles is diverse and varies geographically. A geographic survey across North America by Cato et al. [13] using the standard FAO assay studied PH_3_ resistance in 25 populations of *T. castaneum*. Afful et al. [15] described the distribution of PH_3_ resistance in 34 North American populations of *R. dominica*. Subsequent work is needed on the geographic distribution of phosphine-resistant alleles in resistant populations of the two species to see if any correlation occurs between geographic regions and the resistant alleles for these populations in North America. The objectives of the research described below were to determine the distribution of PH_3_-resistant alleles in the resistant populations of two species of grain beetles, *T. castaneum* and *R. dominica*, using the cleaved amplified polymorphic sequence method, CAPS [23], for specific molecular markers for strong PH_3_ resistance. Additionally, we worked to determine if there were correlations between geographic locations in North America and the distribution of the resistant alleles and/or the resistant phenotypes of the populations for each species.

## 2. Materials and Methods

### 2.1. Insects

One laboratory colony of each species was reared to provide phosphine-susceptible insects for experimental comparisons. These phosphine-susceptible laboratory populations, labeled “Manhattan (LabS)”, were from laboratory colonies maintained in isolation by the USDA Agriculture Research Service laboratory in Manhattan, KS, for more than 50 years and tested regularly with the FAO resistance test [1]. *R. dominica* was reared on a mixture of 95% whole-wheat kernels and 5% admixed cracked kernels (wt:wt), and *T. castaneum* was reared on a mixture of 95% all-purpose wheat flour and 5% Brewer’s yeast (wt:wt). Both species were reared in an incubator at 28 °C and 65% relative humidity with a photoperiod of 16 h light and 8 h dark. This same rearing method was used for short-term colonies of both species, as described below.

Twenty-eight populations of *R. dominica* and 34 populations of *T. castaneum* were used in the study and are shown in Table 1 and Table 2. The approximate locations of the collection site for each population are provided with their corresponding global positioning system (GPS) coordinates for degrees north from the equator and west in Table 1 and Table 2.

### 2.2. Discriminating Dose Bioassay

Prior to our experiments with molecular markers (see methods below), to score genotypes and gene frequencies for a strong resistance allele in phosphine-resistant populations, we needed to estimate the percentage of individuals in each population resistant to phosphine. We, therefore, used a discriminating dose bioassay, known as FAO method No. 16 [24], to test groups of 20 adult beetles from each population to determine the proportion of beetles in a given population with phosphine resistance. We performed the FAO bioassay for 26 out of 28 *R. dominica* populations, which included 7 newly tested populations for the current study and 21 populations that were tested in our earlier work [11,15]. We also used FAO resistance bioassay data for 33 populations of *T. castaneum*, which included 9 newly acquired populations and 24 populations that were tested previously [11,13]. The FAO assays that exposed adult beetles for 20 h, conducted here and in our previous work [11,13,15], used PH_3_ concentrations of 30 ppm for *T. castaneum* and 20 ppm for *R. dominica*. We determined the concentrations of phosphine used in the discriminating doses by quantitative gas chromatography with the same methods used in our earlier work [11,13]. Each group of adult beetles was exposed to a discriminating concentration of phosphine for 20 h at 25 °C, followed by a 14-day period with food in fresh air to allow for either recovery or delayed mortality before assigning an individual as either resistant (alive) or susceptible (dead). We then calculated the percent resistance for each population based on the mortalities.

### 2.3. Generating PCR Markers for PH_3_ Resistant

Cleaved amplified polymorphic sequence (CAPS) markers were used to target a single-nucleotide polymorphism (SNP) found in the DLD gene following the methods described by Chen et al. [11]. We performed CAPS analyses on beetles from 28 populations of *R. dominica*, including 2 populations with all dead individuals preserved in Eppendorf tubes held at −80 °C (populations #10 and #14 in Table 1). Beetles from 34 populations of *T. castaneum* included beetles from one population that had dead individuals also preserved in an Eppendorf tube at −80 °C (population #23 in Table 2). The dead, ethanol-preserved beetles we studied were live-collected from the duff layer of soil surrounding bins with stored wheat at the given location. The living beetles, for molecular marker analyses, were taken from laboratory colonies that started with field collections in 2017 and 2018 and were used in the FAO-discriminating dose bioassay [13,15]. Briefly, a fragment from each of the relevant gene sequences was amplified in a 25 µL reaction volume consisting of 12.5 µL Master Mix, 1 µL each of forward and reverse primers, 2 µL of gDNA template, and 8.5 µL of ddH_2_O using a Thermo Scientific (Waltham MA, USA)PCR MasterMix polymerase kit, as described earlier [11]. Primers, which were the same as described in our earlier work [11], were used in the PCRs for *T. castaneum* and *R. dominica*. The PCR program was set as follows: denaturation at 95 °C for 5 min; 30 cycles at 95 °C for 15 s, 58 °C (55 °C for *R. dominica*) for 30 s, and 72 °C for 2 min for denaturation, annealing, and extension, respectively; and a final extension at 72 °C for 10 min. The amplified 368 bp PCR product from *T. castaneum* and the 375 bp PCR product from *R. dominica* were subjected to separate restriction enzyme digestion with MboI (New England Biolabs, Ipswich, MA, USA) in a 10 µL reaction containing 8 µL of PCR product, 1 µL of reaction buffer, and 1 IU of restriction enzyme. The reaction was then incubated at 37 °C according to the manufacturer’s instructions. We calculated R allele frequencies based on MboI restriction enzyme digestion for each individual in a given population [14].

### 2.4. Statistical Analyses

Four regression analyses using SAS version 8 [25] were conducted for each of the two species from each geographically distributed population sampled, as shown in Table 3. The comparisons via regression were as follows: % resistance via the FAO bioassay vs. the R allele frequency of the population sampled, R allele frequency vs. the latitude of the sampled population, R allele frequency vs. the longitude of the sampled population, and R allele frequency vs. the product of the latitude × longitude for the sampled population. The highest regression values for both species were for % resistance from the bioassay vs. the % frequency of the R allele, and raw data for these were plotted for each of the two species.

## 3. Results

### 3.1. Percentages Resistant to PH_3_ in T. castaneum and R. dominica

PH_3_ resistance measured with the FAO assay was determined in six new populations of *R. dominica* beyond those reported in 2018 [15]. These included two populations from Kansas, one from Oklahoma, one from Arkansas, and two from Georgia, showing a range of 0% to 97% resistance in the bioassay (Table 1). There were nine newly tested populations of *T. castaneum*, including one from Arizona, four from Kansas, one from Oklahoma, one from Illinois, one from Alabama, and one from Kentucky, which were found to have resistance values ranging from 0% to 95% in the bioassay (Table 2).

### 3.2. R Allele Frequencies in the Populations of T. castaneum and R. dominica

**Figure 1:**
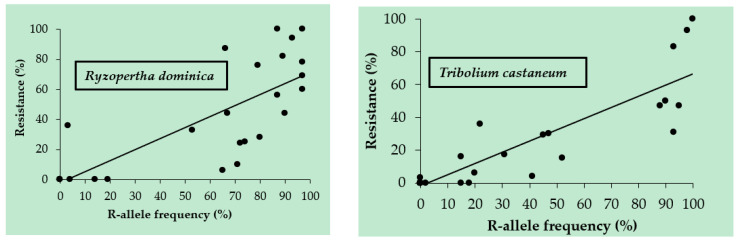
Plots of percentages of phosphine-resistant beetles found using the FAO bioassay and the frequency of the strong phosphine resistance allele in geographically separate populations of the lesser grain borer, *Rhyzopertha dominica* (**left**), and the red flour beetle, *Tribolium castaneum* (**right**).

Molecular marker analyses for 28 populations of *R. dominica*, including 2 populations analyzed from preserved individuals (populations 10 and 14 in Table 1), and 34 populations of *T. castaneum*, including 1 population analyzed from preserved individuals (population 23 in Table 2), were carried out based on DLD mutations at P45S in *T. castaneum* and P49S in *R. dominica* [11]. High R allele frequencies were observed in populations with high levels of survivorship in the FAO-discriminating dose bioassays in both species (Table 1 and Table 2). Regression analyses between the percentage resistant to PH_3_ from the FAO bioassays and R allele frequencies in both species resulted in R^2^ values of 0.59 for *R. dominica* and 0.79 for *T. castaneum*, as reported in Table 3 and shown in Figure 1. R allele frequencies of the sampled populations for both species had low R^2^ values at extremely low F-statistics when compared to latitude, longitude, and the products of both latitude and longitude for each population (Table 3).

## 4. Discussion

We showed that the phenotype of phosphine resistance measured by the FAO-discriminating dose bioassay was highly correlated with the R allele frequency for PH_3_ resistance in North American populations of *R. dominica* and *T. castaneum*. The data suggest that the higher survivorship in the bioassay is associated with higher R allele frequency, as we reported in our previous study [11]. In that study, we reported that the strongly resistant populations of *T. castaneum* were correlated with R allele frequency after we analyzed seven populations of this species collected from Kansas, USA. We extended our collections of these two species to 28 populations from eight states of the USA and two provinces of Canada for *R. dominica* and 34 populations from twelve states of the USA and four provinces of Canada for *T. castaneum* (Table 1 and Table 2). The majority of the populations studied here by CAPS marker analysis were previously reported as being resistant to phosphine gas [11,13,15]. The results of the current study show that there are much higher frequencies of PH_3_-resistant insects in the US compared to those in the first global geographic study conducted by Champ and Dyte [1]. They found that the three populations of *R. dominica* in North America were susceptible to PH_3_, whereas only one out of seventeen populations of *T. castaneum* was resistant to PH_3_ at that time. The same study reported resistance occurring at frequencies ranging from 11 to 57% in *R. dominica* across the remaining seven continental regions.

The results of our FAO bioassay study showed that 11 populations out of 34 of *T. castaneum* and 3 populations out of 28 of *R. dominica* were totally susceptible to PH_3_ with zero R allele frequency (Table 1 and Table 2). These results could suggest that the evolution of PH_3_ resistance in *R. dominica* was much faster than that in *T. castaneum*, as described in Opit et al. [8]. They found that the percentage that was PH_3_-resistant in North American populations of *R. dominica* increased from 69% in 1990 to 100% in 2012, whereas resistance in North American populations of *T. castaneum* increased from 13% in 1990 to 89% in 2012 [3,8]. This difference in PH_3_ resistance in these two storage pest insects is also reflected in the results of the current study from Canada (Table 1 and Table 2). Three out of four populations of *R. dominica* were weakly resistant to PH_3_ with zero R frequency, whereas all populations of *T. castaneum* were susceptible to PH_3_. In addition, the weak resistance in three out of four populations of *R. dominica* in Canada indicates that it is caused by mutation(s) in the *rph1* gene, a cytochrome b5 fatty acid desaturase (*Cyt-b5-r*) that apparently codes for the weak resistance phenotype in this grain pest [26]. In addition, the percentage of resistance in three out of eight populations of *R. dominica* in Canada was more than 50% [15]. CAPS marker analysis was not conducted for the three resistant populations of *R. dominica* from Canada because the samples were unavailable when we conducted the CAPS marker analyses in the present study. Therefore, future research on the gene mutation of *rph1* and rph2 in these weakly and relatively strongly resistant populations of *R. dominica* from Canada would be interesting to pursue. Previous studies [13,15] and the current study show that the low-resistance frequencies in these two beetle species in Canada, especially in western parts of Ontario, were most likely caused by low selection pressure for resistance. The cooler climate in Canada compared to that of the USA likely results in lower insect infestation rates and, therefore, less frequent phosphine fumigations for pest control in most years (unpublished reports from the Canadian Grain Commission).

Our research found no substantial correlation between the R allele frequency for phosphine resistance in a given population and its geographic distribution in either of these serious grain storage pest species. These results further confirm that the evolution of PH_3_ resistance in these two species was likely due more to selection pressure from frequent fumigations instead of high levels of gene flow based on proximity to resistant conspecifics.

In conclusion, PH_3_-resistant populations of two serious grain storage pests, *T. castaneum* and *R. dominica*, are widely distributed throughout North America. The PH_3_ resistance levels in these populations in each species are more correlated with the frequency of a gene for phosphine resistance across populations, but PH_3_ resistance was less correlated with the geographic locations of the tested populations.

## Figures and Tables

**Table 1:** Frequency of resistance in populations of *Rhyzopetha dominica* from specific locations as determined by the discriminating dose bioassay, along with the frequency of the strong R allele in that population.

Population #	Locality		GPS Coordinates	% Resistance via Bioassay	% R Allele Frequency
	**State/Province**	**City ^1^**			**(*n*)**
1	CA	Princeton #	N39.24W122.03	71	10 (21)
2	CA	Williams #	N39.92W122.86	53	33 (16)
3	CA	Colusa #	N39.21W122.01	3	36 (18)
4	CA	Parlier-1 #	N36.36W119.31	97	60 (16)
5	CA	Parlier-2 #	N36.36W119.31	72	24 (18)
6	KS	Manhattan (LabS)	N39.11W96.34	0	0 (16)
7	KS	Abilene-1 #	N38.55W97.11	93	94 (16)
8	KS	Garden City #	N37.58W97.13	74	25 (16)
9	KS	Abilene-2 #	N38.53W97.13	90	44 (16)
10	KS	Hudson *	N38.62W98.39		65 (12)
11	KS	Clifton #	N39.34W97.16	80	28 (16)
12	KS	Junction City	N39.15W100.52	97	100 (16)
13	KS	Manhattan USDA	N39.11W96.34	66	87 (16)
14	KS	Konza *	N39.92W96.35		3 (15)
15	OK	Stillwater	N36.66W97.33	65	6 (16)
16	OK	Garfield $	N36.26W97.52	97	69 (16)
17	OK	Logan $	N36.34W100.13	90	78 (16)
18	TX	Victoria #	N28.48W97.01	67	44 (16)
19	TX	Burleson #	N32.32W97.19	87	56 (16)
20	AR	Jonesboro	N35.50W90.42	79	76 (16)
21	AL	Uniontown #	N32.27W87.31	89	82 (16)
22	GA	Tifton	N31.27W83.30	0	0 (16)
23	GA	Nashville	N31.12W83.15	0	0 (16)
24	FL	Belle Glade #	N26.41W80.40	87	100 (16)
25	Alberta	Lethbridge #	N49.41W112.50	4	0 (18)
26	Alberta	Stirling #	N49.31W112.31	14	0 (18)
27	Saskatchewan	Carnduff #	N49.10W101.47	0	0 (18)
28	Saskatchewan	Coronach #	N49.65W105.31	19	0 (18)

^1^ Populations for which ethanol-preserved adults were analyzed for their R allele frequency, but those for which no resistance bioassays were performed are marked with “*”; bioassay data from Afful et al. 2018 [15] are marked with “#”, and those from Chen et al. 2015 [11] are marked with “$”.

**Table 2:** Frequency of resistance in populations of *Tribolium castaneum* from specific locations as determined by a discriminating dose bioassay, along with the frequency of the strong R allele in that population.

Population #	Locality		GPS Coordinates	% Resistance via Bioassay	% R Allele Frequency
	**State/Province**	**City ^1^**			**(*n*)**
1	CA	Williams #	N.39.09W122.09	0	0 (18)
2	CA	Arbuckle #	N39.01W122.03	0	0 (17)
3	CA	Davis #	N38.32W121.44	0	0 (18)
4	AZ	Arizona	N/A	0	0 (16)
5	KS	Manhattan (LabS)	N39.11W96.34	0 #	0 (16)
6	KS	Washington #	N39.50W97.03	15	0 (16)
7	KS	Minneapolis #	N39.07W97.42	93	83 (16)
8	KS	Mitchel	N38.23W98.06	95	47 (16)
9	KS	Russell	N38.53W98.50	45	29 (16)
10	KS	McPherson	N38.22W97.40	47	30 (16)
11	KS	Abilene-1 #	N38.55W97.11	41	4 (16)
12	KS	Abilene-2	N38.53W97.13	52	15 (16)
13	KS	Manhattan USDA #	N39.11W96.34	15	16 (16)
14	OK	Garfield $	N36.26W97.52	90	50 (16)
15	OK	Logan $	N36.34W100.13	93	31 (16)
16	OK	Stillwater $	N36.66W97.33	18	0 (16)
17	OK	Miami	N36.52W94.52	0	0 (16)
18	TX	Victoria #	N28.48W97.01	2	0 (18)
19	IL	Chicago	N41.51W87.39	0	0 (16)
20	MO	Excelsior Springs #	N39.20W94.13	0	0 (18)
21	AR	Jonesboro #	N35.50W90.42	20	6 (16)
22	AL	Centre	N34.10W85.40	0	3 (16)
23	AL	Pratville *	N32.28W86.27		16 (16)
24	AL	Uniontown #	N32.27W87.31	31	17 (14)
25	AL	Red Level #	N31.18W86.30	100	100 (16)
26	AL	Ozark #	N31.27W85.38	98	93 (16)
27	KY	HI KY	N/A	0	0 (16)
28	GA	Nashville #	N31.12W83.15	22	36 (22)
29	GA	Tifton #	N31.27W83.30	0	0 (16)
30	FL	Walnut Hill #	N30.54W87.30	88	47(17)
31	Alberta	Calgary #	N51.2W114.03	0	0 (16)
32	Saskatchewan	Saskatoon #	N52.8W106.40	0	0 (16)
33	Manitoba	Winnipeg #	N49.54W97.08	0	0 (16)
34	Quebec	St. Agathe #	N46.23W71.24	0	0 (16)

^1^ Ethanol-preserved adults were analyzed for their R-allele frequency, but those for which no resistance bioassays were performed are marked with “*”; bioassay data from Cato et al. 2017 [13] are marked with “#”, and those from Chen et al. 2015 [11] are marked with “$”.

**Table 3:** Regression analyses for the frequency of the R-allele gene for strong resistance to phosphine, the phosphine resistance phenotype, and geographic location variables for North American populations of *Rhyzopertha dominica* and *Tribolium castaneum*.

Species	Variables	Equation	R^2^	F-Statistic	*p*-Value
*R. dominica*	% resistance vs. R-allele frequency	y = 0.7329x – 1.85	0.592	0.810044	<0.001
	R-allele frequency vs. Latitude	y = −2.6476x + 140.82	0.203	5.15863 × 10^−13^	0.361
	R-allele frequency vs. Longitude	y = −0.9637x + 138.86	0.121	2.68436 × 10^−6^	<0.001
	R-allele frequency vs. Latitude × Longitude	y = −0.0169x + 107.85	0.205	1.49053 × 10^−28^	<0.001
*T. castaneum*	% resistance vs. R-allele frequency	y = 0.6798x − 1.53	0.787	0.143403	<0.001
	R-allele frequency vs. Latitude	y = −1.8806x + 91.10	0.147	3.26431 ×10^−9^	0.002
	R-allele frequency vs. Longitude	y = −0.6448x + 82.00	0.068	3.60977 × 10^−6^	<0.001
	R-allele frequency vs. Latitude × Longitude	y = −0.0129x + 67.23	0.146	2.48567 × 10^−41^	<0.001

## Data Availability

The original contributions presented in this study are included in the article. Further inquiries can be directed to the corresponding author.
